# Meeting of Southern Medical Association

**Published:** 1915-11

**Authors:** 


### Meeting of Southern Medical Association.

The following special letter from Dr. Oscar M. Marchman of the membership committee of the Southern Medical Association should bring hearty co-operation from every member of the medical profession in Texas. The men who fail to attend the meeting of the Southern Medical Association will lose an opportunity of a lifetime. Let us all go, and wear our best clothes, our happiest smiles, and thereby convince the doctors of the United States that we know how to entertain and how to be entertained.—EDITOR.

*To Medical Men of Texas and the South, Friends All:*

Now as never before has opportunity and its consequent responsibility combined to challenge our progressive spirit and professional patriotism.

During the years the balance of scientific credit has apparently been in favor of the East and North, probably because of the traditional belief that we must look upward for inspiration and to the eastern horizon for intellectual light as for physical illumination.

We of the South, seconded by our friends of the West, have wanted a way whereby the land of Crofford Long, Ephraim McDowell, Marian Sims, and other illustrious pioneers, might prove its title to sustained leadership in the march of progressive evolution.

It was in response somewhat to such an ambition that the Southern Medical Association was organized, and happily it is giving voice to the collective learning and clinical experience of the men who are making the up-to-date medical record of the South, thus making these forceful factors available for the common good, after passing the clearing house of Friendly Criticism, such as attends full, free, and frank discussions.

These facts, essentially pertinent as relating to the South as a whole, apply with immediate acuteness to Texas and its nearest neighbors, and to every conscientious practitioner in this territory, we especially appeal for a fraternal co-operation of the very highest order.

Every physician who is eligible to membership should consider it a duty and a privilege to be identified with the Southern Medical Association, because it is performing a splendid service to the profession that benefits every medical man in the region from which its membership is drawn.

We now have a membership of over five thousand of the most progressive physicians in the sixteen Southern States. The enthusiasm of its members shows that Southern physicians realize their opportunities; that they propose to do their part in the solution of health problems that affect Southern prosperity, and that they intend to aid in the great movement for the advancement of Southern medicine and surgery.

The great Texas meeting will be held in Dallas, November 8, 9, 10, and 11. Southern Medical invites you. Dallas invites you to take part in the greatest medical event which Texas has ever known.

SPECIAL REQUEST.

Let every devotee to regular medicine reading this "Call to Arms" immediately write to some Dallas doctor of his acquaintance, or to the president of the Dallas County Medical Society, indicating the probability of his being here in November. Such information when compiled will aid us in knowing the extent of preparation, general and special, including the big banquet to which each of you are invited with infinite cordiality. Also write to a friend or friends to meet you; this will stimulate attendance and make reunions a delightful feature.

Write these letters promptly. "Clean your spark-plugs." "Adjust your carburetor," and if your "self-starter won't work," get the good wife to pilot you, for she, too, will find the glad hand in warmest welcome.

The solid South for a better profession.

Yours for two thousand new members,

MEMBERSHIP COMMITTEE.

---

(Tune of "Tipperary.")

In November, please remember, come to Dallas town;
We're preparing stunts for you, the best that can be found.
We want every Texas doctor lined up right away,
In November be a member of the S. M. A.

CHORUS.

It's a fine time you'll have in Dallas,
It's a short trip to go,
You will meet there all the "Big Ones"
That you'd surely like to know.
We will greet you when we meet you,
In the good old Texas way:
So be sure to come and visit Dallas
And join the S. M. A.

Well, boys, it's up to Dallas,
They're a-coming here soon;
You know what we promised
When we "bulled" them last June—
Two thousand new members,
If they'd meet here in the fall.
No "pie-crust" goes with that promise,
We've got to get them, that's all.

The fact that the finance committee states that approximately $6000 will be spent on entertainment proves conclusively that there will be nothing left undone in the way of amusement and entertainment for the visitors. Below is given an outline of the social program:

The general outline of the social program of the convention is as follows:

Sunday Afternoon—3 to 5 o'clock, sacred concert, Scottish Rite Cathedral.

Sunday Night—8 o'clock, health sermons at various churches.

Monday Afternoon—3 o'clock, automobile ride for ladies.

Monday Night—8 o'clock, health lecture; 10 to 12, smoker, palm garden, Adolphus.

Tuesday Afternoon—4 to 6 o'clock, musicale for ladies, Dallas Golf and Country Club; Dallas ladies to be assisted by wives of Fort Worth doctors.

Tuesday Night—8 o'clock, health lecture; 9:30 to 12 o'clock, reception, president and guests, dancing, Scottish Rite Cathedral, Fort Worth doctors and wives to be in receiving line.

Wednesday Afternoon—4 to 6 o'clock, reception for ladies, Lakewood Club, ladies to be assisted by wives of Fort Worth doctors.

Wednesday Night—7:30 o'clock, banquet and short speeches, Scottish Rite Cathedral.

All business sessions will be held in the auditorium of the city hall.

---

Dr. John O. McReynolds, chairman of the committee on alumni reunions for the Dallas meeting of the Southern Medical Association, is anxious to secure the active co-operation of all alumni associations that may be represented at this meeting. He urgently requests the officers of the various alumni who will be present to communicate with him at once.

